# Insights from monkey malaria that can change thinking about human infections

**DOI:** 10.1186/1475-2875-9-S2-I2

**Published:** 2010-10-20

**Authors:** Janet Cox-Singh

**Affiliations:** 1 Centre for Infection, St George's University of London, London, UK

## 

Despite prolonged and intense effort to understand and control the health impact of malaria morbidity and mortality, malaria remains third in the global ranking for severe and fatal infections. *P. falciparum* is responsible for most malaria morbidity and mortality [1]. The more widespread *Plasmodium vivax* is considered less pathogenic but now appears to cause severe acute disease with some fatal cases [2]. Nevertheless among the human malarias virulent acute *P. falciparum* malaria attracts most research attention.

Falciparum malaria has several characteristics notfound in *P. vivax* or the other two human adapted species, *Plasmodium ovale* and *Plasmodium malariae.* Differences include parasitemias >100 000/uL and the ability to sequester from the peripheral blood in deep post-capillary venules. Both are strong contenders for increased pathophysiology in this species. Unfortunately because of few similarities between the four human-host malarias it is difficult distinguish virulence factors from interspecies differentiation.

The monkey malaria, *Plasmodium knowlesi* may change this and our views on malaria pathophysiology. The discovery of severe and fatal cases of knowlesi malaria in the human population was unexpected and for the first time offers a comparator disease for falciparum malaria. In Sarawak, Malaysian Borneo 10% of *P. knowlesi* cases are complicated, with at least 1% of all human cases becoming fatal [3]. Complicated knowlesi malaria presents as organ dysfunction, acute and delayed acute respiratory distress syndrome, but not coma or severe malarial anaemia that are often associated with falciparum malaria. Preliminary studies show a direct correlation between *P. knowlesi* parasitemia and complications, parasitemias >100 000/uL are common. Blood vessels in the brain from a fatal knowlesi case were congested with sequestered heavily pigmented mature stage parasites [4]. The patient did not present with cerebral malaria, a complication often associated with *P. falciparum* sequestration in post capillaries of the brain.

Descriptions of naturally acquired *P. knowlesi* malaria in humans are enhanced by the wealth of baseline molecular information available for *P. knowlesi* gathered from experimental primate infections and the knowlesi genome project [5]. *P. knowlesi* is permissive in various primates and some infections are representative of human knowlesi malaria. Pathophysiology of naturally acquired *P. knowlesi* malaria in humans and *P. falciparum* compare well and there are important differences. Comparisons will lead to the identification of testable candidate virulence factors. The ability to specifically test virulence in representative primate models may well offer much needed means to understand malaria pathophysiology and to help distinguish *P. falciparum* virulence from inconsequential species-specific characteristics.
